# Storage nitrogen co-ordinates leaf expansion and photosynthetic capacity in winter oilseed rape

**DOI:** 10.1093/jxb/ery134

**Published:** 2018-04-12

**Authors:** Tao Liu, Tao Ren, Philip J White, Rihuan Cong, Jianwei Lu

**Affiliations:** 1 Microelement Research Center, Huazhong Agricultural University, Wuhan, China; 2 The James Hutton Institute, Invergowrie, Dundee, UK

**Keywords:** *Brassica napus* L, leaf expansion, nitrogen partitioning, photosynthesis, storage nitrogen, storage nitrogen form

## Abstract

Storage nitrogen (N) is a buffer pool for maintaining leaf growth and synthesizing photosynthetic proteins, but the dynamics of its forms within the life cycle of a single leaf and how it is influenced by N supply remain poorly understood. A field experiment was conducted to estimate the influence of N supply on leaf growth, photosynthetic characteristics, and N partitioning inthe sixth leaf of winter oilseed rape (*Brassica napus* L.) from emergence through senescence. Storage N content (*N*_store_) decreased gradually along with leaf expansion. The relative growth rate based on leaf area (*RGR*_a_) was positively correlated with *N*_store_ during leaf expansion. The water-soluble protein form of storage N was the main N source for leaf expansion. After the leaves fully expanded, the net photosynthetic rate (*A*_n_) followed a linear–plateau response to *N*_store_, with *A*_n_ stabilizing at the highest value above a threshold and declining below the threshold. Non-protein and SDS (detergent)-soluble protein forms of storage N were the main N sources for maintaining photosynthesis. For the leaf N economy, storage N is used for co-ordinating leaf expansion and photosynthetic capacity. N supply can improve *N*_store_, thereby promoting leaf growth and biomass.

## Teaser

Optimal storage nitrogen (N) content in the leaf is critical to co-ordinate leaf expansion and photosynthetic capacity. Turnover between storage N and photosynthetic N could maintain a high photosynthetic rate.

## Introduction

As a major mineral nutrient that limits the growth of plants, nitrogen (N) absorption by plants is always faster than is required for their current growth, resulting in N accumulation in the tissue and the formation of N reserves (Millard, 1988). The stored N can act as a buffer pool for asynchrony between N supply and demand for growth, and N storage is a predominant strategy for the efficient utilization when suffering from potentially limited N (Bloom *et al.*, 1985; Staswick, 1994; Rossato *et al.*, 2001).

Storage N is a buffer pool for maintaining leaf expansion and synthesizing photosynthetic proteins in early leaf growth (Chapin *et al.*, 1990; Lehmeier *et al.*, 2013). In the initial growth stage of leaves, both the formation of the photosynthetic apparatus and the expansion of leaves require a large quantity of N (Evans, 1989; Johnson *et al.*, 2010), leading to competition for N between cell division and chloroplast development (Miyazawa *et al.*, 2003; Kusumi *et al.*, 2010). Under N deficiency, cell division and enlargement would be limited, affecting the final leaf area (Roggatz *et al.*, 1999). However, there is variation among species under N deficiency. For example, potato and maize have two entirely different strategies in response to N deficiency (Hirel *et al.*, 2007). At the single-leaf level, potato maintains leaf N concentration and photosynthetic capacity by reducing the leaf size (Vos and Van der Putten, 1998), whereas maize shows a tendency to expand leaf size, resulting in a decline in leaf N concentration and photosynthetic capacity (Vos *et al.*, 2005). Furthermore, the duration of the high photosynthetic rate is the key to improving photosynthetic productivity after the leaf is fully expanded (Makino *et al.*, 1984; Richards, 2000). Thomas and Howarth (2000) have shown that if leaf senescence is delayed for 2 d, fixed carbon will be increased by ~11%. Increasing the N supply could maintain the photosynthetic protein content in leaves, delaying the onset of senescence (Osaki, 1995). In mature leaves, N influx decreased sharply, almost in equilibrium with N efflux (Makino *et al.*, 1984; Imai *et al.*, 2005), indicating that the leaves had to maintain photosynthetic capacity using their own reserves of N. Moreover, the N accumulation in leaves was in excess of the sum of structural and photosynthetic N (Pask *et al.*, 2012). Therefore, storage N is mainly used for maintenance of photosynthetic metabolism after the leaves fully expanded. However, it is essential to clarify the relationships between storage N and leaf expansion/photosynthesis to understand functionally the mechanisms of storage N for balancing leaf expansion and photosynthetic capacity.

Storage N in leaves is mainly in the forms of nitrate, amino acid, and protein (Millard, 1988; Nordin and Näsholm, 1997; Tegeder *et al.*, 2018). Nitrate in leaves is mostly stored in vacuoles, accounting for 58–99% of total leaf nitrate (Martinoia *et al.*, 1981; Granstedt and Huffaker, 1982). Nitrate stored in vacuoles not only drives cell expansion but also affects the rate and duration of leaf expansion (Sprent and Thomas, 1984; Armstrong *et al.*, 1986). Under N limitation, nitrate could be discharged into the cytoplasm in mature leaves. The concentrations of free amino acids in leaves are closely related to leaf growth (Masclaux *et al.*, 2000; Havé *et al.*, 2016). Increased N supply significantly increases the concentration of free amino acids in leaves (Lemaître *et al.*, 2008; Clément *et al.*, 2017). Proline, glutamine, and arginine can be used as N storage sources, and their use varies by species (Nordin and Näsholm, 1997; Lemaître *et al.*, 2008). Most of the N stored in leaves is stored as protein. There is a widespread consensus that vegetative storage protein (VSP) is specifically used for temporary storage of N (Langheinrich,1993; Rossato *et al.*, 2001, 2002; Lee *et al.*, 2014). Nevertheless, it would be accumulated in senescent leaves, rather than in mature leaves. Moreover, the accumulation of VSP is always concomitant with the remobilization of both subunits of Rubisco. Therefore, the relationship between storage N and leaf growth can be better understood by exploring the distribution and changes in different types of storage N.

Although nitrate, free amino acids, and VSP are important components of storage N in leaves, they are only a small proportion of the ‘storage N pool’ and cannot explain all the storage N in leaves. Broadly speaking, leaf N that is not involved in any metabolic process can be attributed to storage N (Xu *et al.*, 2012). Takashima *et al.* (2004) divided leaf N into four fractions as follows: detergent-insoluble proteins, detergent-soluble proteins, water-soluble proteins, and other N. The chemical extraction method can distinguish the different forms of N, but the N stored in each form cannot be distinguished. Based on the leaf utilization of N assimilation model (LUNA) (Xu *et al.*, 2012; Ali *et al.*, 2016), leaf N can be divided into four fractional pools as follows: structural N, respiratory N, photosynthetic N, and storage N. Although the model can roughly estimate the total amount of storage N, it cannot distinguish the different forms of storage N.

Winter oilseed rape (*Brassica napus* L.) is characterized by a high N requirement and low N fertilizer use efficiency (Schjoerring *et al.*, 1995; Hirel *et al.*, 2007; Avice and Etienne, 2014; Bouchet *et al.*, 2016). In its characteristic N absorption pattern, the biomass at the seedling stage accounts for only 20–30% of that over the whole growth period, but N is accumulated to ~80% of the maximum (Barraclough, 1989; Bouchet *et al.*, 2016; Li *et al.*, 2016). Most of the N is accumulated in the leaves; the N content based on mass in the leaves is even as high as 7% at the seedling stage (Malagoli *et al.*, 2005), resulting in a considerable proportion of N as storage N. Moreover, the life span of leaves is long at 30–105 d; and the rates of leaf emergence and expansion are relatively slow at the seedling stage (Jullien *et al.*, 2011). Therefore, winter oilseed rape is a good material for studying storage N. Previous studies have found that there are large amounts of amino acids (Tilsner *et al.*, 2005; Lemaître *et al.*, 2008) and VSP (Rossato *et al.*, 2001, 2002) in oilseed rape leaves, but far more N than that is stored in the leaves. Here we combined the model estimation with the chemical method to clarify the relationship between the functions and forms of N. In particular, storage N was divided into three types and quantitatively analysed. Therefore, this study had the following objectives: (i) to clarify the relationship between storage N and leaf expansion and photosynthetic capacity and (ii) to quantify the contribution of different forms of storage N during leaf growth. To achieve these objectives, this study was conducted using winter oilseed rape with different levels of N supply to investigate the distribution of photosynthetic N and storage N within the life cycle of a single leaf at the seedling stage.

## Materials and methods

### Site characteristics

**Fig. 1.**
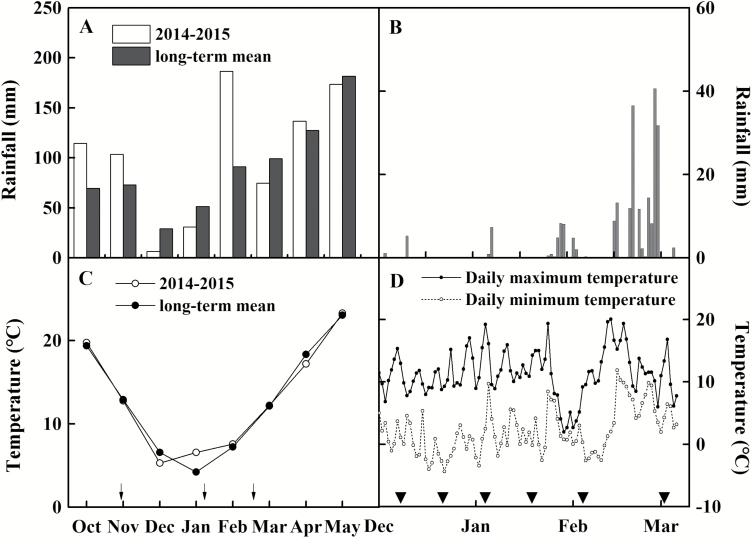
The rainfall and temperature during the winter oilseed rape growing season (A, C) and the experimental period (B, D; December 2014–March 2015). Inverted filled triangles indicate the timing of gas exchange and sampling time: 5–7 December 2014 (S1), 19–21 December 2014 (S2), 3–5 January 2015 (S3), 16–18 January 2015 (S4), 5–7 February 2015 (S5), 2–4 March 2015 (S6). Black arrows indicate the timing of fertilization.

The field experiment was carried out from September 2014 to May 2015 in Wuxue County (30°06'47''N, 115°35'35''E), Hubei Province, central China. The experimental site was located in the subtropical monsoon climate zone. The soil properties in the 0–20 cm soil layer were as follows: pH, 5.60 (suspension of 1 g soil in 5 cm^3^ water); organic matter, 29.69 g kg^–1^; total N, 1.69 g kg^–1^; Olsen-P, 8.10 mg kg^–1^; NH_4_OAc-K, 47.21 mg kg^–1^; hot water-soluble B, 0.75 mg kg^–1^. The rainfall and temperature during the growing season of winter oilseed rape and the time period of the experiment (from December to March) are shown in Fig. 1. The monthly rainfall and temperature were close to the long-term mean, except for significantly higher rainfall in February 2015, which was concentrated in late February.

### Experimental design

The experiment was conducted using a complete randomized block design with three replicates, which included treatments with four amounts of N fertilizer as follows: (i) N_0_, without N fertilization treatment; (ii) N_90_, N fertilizer applied at 90 kg N ha^–1^; (iii) N_180_, N fertilizer applied at 180 kg N ha^–1^; and (iv) N_360_, N fertilizer applied at 360 kg N ha^–1^. Each individual plot was 20 m^2^ in area.

Except for N fertilizer, the other fertilizers, phosphate (P), potassium (K), and boron (B), were the same for all the treatments: 90 kg P_2_O_5_ ha^–1^, 120 kg K_2_O ha^–1^, and 1.6 kg B ha^–1^. The application rates were recommended by local agricultural technicians and were sufficient to meet the requirements of winter oilseed rape. The fertilizer types used in the study were urea (46% N), calcium superphosphate (12% P_2_O_5_), potassium chloride (60% K_2_O), and borax (10.8% B). N fertilizer (urea) was applied in three splits: 60% for basal fertilizer (BBCH 15–16; 30 October 2014) (Lancashire *et al.*, 1991), 20% for top dressing at the overwintering stage (BBCH 29; 6 January 2015), and 20% for top dressing at the initiation of stem elongation (BBCH 30; 13 February 2015) (Fig. 1). All P, K, and B fertilizers were applied as base fertilizers.

The cultivar was Huayouza No. 9, which is grown widely in the middle reaches of the Yangtze River. Winter oilseed rape was seeded on 24 September 2014 and, at 37 d after sowing (BBCH 15–16; 31 October 2014), uniform seedlings with 4–5 leaves were selected and transplanted to the plots by hand, with a transplanting density of 1.125 × 10^5^ plants ha^–1^ (225 plants per plot). Weeds, pests, and diseases were controlled using chemical control, following local methods. No obvious weed, pest, or disease problems occurred during the growing season of winter oilseed rape.

### Sampling and measurements

#### Leaf tagging

Thirty days after transplanting (30 November 2014), each plant had a total of 5–6 leaves, with no difference between N treatments. Forty uniform plants per plot were selected for marking of the newly appeared sixth leaf in order to monitor leaf growth. From the time of marking, four of the marked leaves per plot were selected for determination of photosynthetic parameters approximately every 2 weeks (see Fig. 1 for the specific sampling time, and Supplementry Fig. S1 at *JXB* online for pictures during leaf growth). After measurement of photosynthetic parameters, the physiological parameters of the selected leaves, including leaf area, dry matter, chlorophyll content, and contents of total N and different forms of N were determined.

#### Photosynthetic parameters

Between 10:00 am and 15:00 pm, selected leaves were measured for net photosynthetic rate (*A*_n_, μmol m^–2^ s^–1^) and *A*/*C*_i_ response curves using a LI-6400XT portable photosynthesis open system (LI-COR Biosciences, Lincoln, NE, USA), equipped with a CO_2_ control module and a red–blue LED light source. The photosynthetic photon flux density (PPFD) of the leaf chamber was set to 1200 μmol m^–2^ s^–1^ (with 90% red light and 10% blue light). The leaf chamber temperature and the air flow rate were set as 15 °C and 500 μmol s^–1^, respectively. The reference chamber CO_2_ concentration was set at 400 μmol mol^–1^ for *A*_n_ measurement; for measurement of the *A*/*C*_i_ response curves, it was adjusted based on the following series: 400, 300, 200, 150, 100, 50, 400, 400, 600, 800, 1000, 1200, and 1500 μmol mol^–1^. The maximum carboxylation rate (*V*_c,max_, μmol CO_2_ m^–2^ s^–^) and the maximum electron transport rate (*J*_max_, μmol e^–^ m^–2^ s^–1^) were calculated using the *A*/*C*_i_ curve according to the Long and Bernacchi (2003) method. The details are as follows:

$$A_{n}\;=V_{c,max}\frac{C_{i}-\Gamma^{*}}{C_{i}+K_{c}(1+O/K_{o})}-R_{d}$$ (1)

$$A_{n}=J_{max}\frac{C_{i}-\Gamma^{*}}{4C_{i}+8\Gamma^{*}}-R_{d}$$ (2)

where *C*_i_ is the intercellular CO_2_ concentration (μmol mol^−1^), Γ* is the CO_2_ compensation point (μmol mol^−1^), *K*_c_ and *K*_o_ are Michaelis constants for carboxylation and oxygenation, *O* is the O_2_ concentration (210 000 μmol mol^−1^), and *R*_d_ is the mitochondrial respiration rate in the light (μmol CO_2_ m^–2^ s^–1^). Additionally, *K*_c_ and *K*_o_ calculated by the temperature dependence function (Bernacchi *et al.*, 2001) are 167 290 μmol mol^−1^ and 133 μmol mol^−1^, respectively, at 15 °C (for details, see Supplementary Text S1).

### Leaf area, relative growth rate, and chlorophyll concentration

After the determination of the photosynthetic parameters, the measured leaves were stored in a freezer until transferred to the laboratory. The leaves with the main veins removed were placed on A3 paper, and a 5 × 5 cm piece of green cardboard was added as a control. Images were obtained with a digital camera (D700, Nikon, Inc., Japan), and Image-Pro Plus 6.0 software (Media Cybernetics, Silver Spring, MD, USA) was used to obtain the leaf area (Abràmoff *et al.*, 2004). Relative growth rate was calculated based on leaf area (*RGR*_a_, cm^2^ cm^−2^ d^−1^):

$$RGR_{a}\;=\frac{1}{Q}\times \frac{dQ}{dt}$$ (3)

where *Q* is the leaf area and d*Q*/d*t* is the transient increment.

From the middle parts of the leaves (i.e. the photosynthetic measurement position), leaf discs were created with a 10 mm diameter punch. Three of them were weighed and then stored at –80 °C. After sampling, the rest of each leaf was weighed, placed at 105 °C for 30 min, and then dried at 60 °C to constant weight. One leaf disc was prepared for analysis of the chlorophyll concentration using Porra’s method (Porra *et al.*, 1989). The leaf discs were extracted with 50 ml of 80% acetone, soaked for 48 h. The concentrations of Chl *a* and *b* were measured at 663.8 nm and 646.8 nm, respectively, using a spectrophotometer (UV2102 PCS, Unico, China).

### N partitioning by morphology

Different forms of N were measured from the frozen leaf discs using the method of Takashima *et al.* (2004) with minor modifications. The leaves were powdered in liquid N and homogenized with 1 ml of 100 mM Na phosphate buffer (pH 7.5 and containing 0.4 M d-sorbitol, 2 mM MgCl_2_, 10 mM NaCl, 5 mM iodoacetate, 5 mM phenylmethylsulphonyl fluoride, and 5 mM DTT), then washed in a centrifuge tube, and this was repeated four times. The supernatant (regarded as water-soluble protein, *N*_w_) was separated by centrifugation at 15 000 *g*, 4 °C for 15 min. Then, 1 ml of phosphate buffer containing 3% SDS was added to the pellet, followed by heating in 90 °C water for 5 min. The mixture was centrifuged at 4500 *g* for 10 min. This procedure was repeated six times, while the supernatants were collected (regarded as the SDS-soluble protein, *N*_s_). The residue (regarded as the SDS-insoluble protein, *N*_in-SDS_) was washed with ethanol into the quantitative filter paper. The supernatant was added to an equal volume of 20% trichloroacetic acid to denature the protein, after which it was filtered with quantitative filter paper and washed with ethanol. The three types of components of N on the quantitative filter paper were dried and digested with H_2_SO_4_–H_2_O_2_ by the method of Thomas *et al.* (1967). The N concentration in the digestion solution was analysed by continuous flow analysis (AA3, Seal Analytical Inc., Southampton, UK). The same quantitative filter paper without samples was used as a control.

**Fig. 2.**
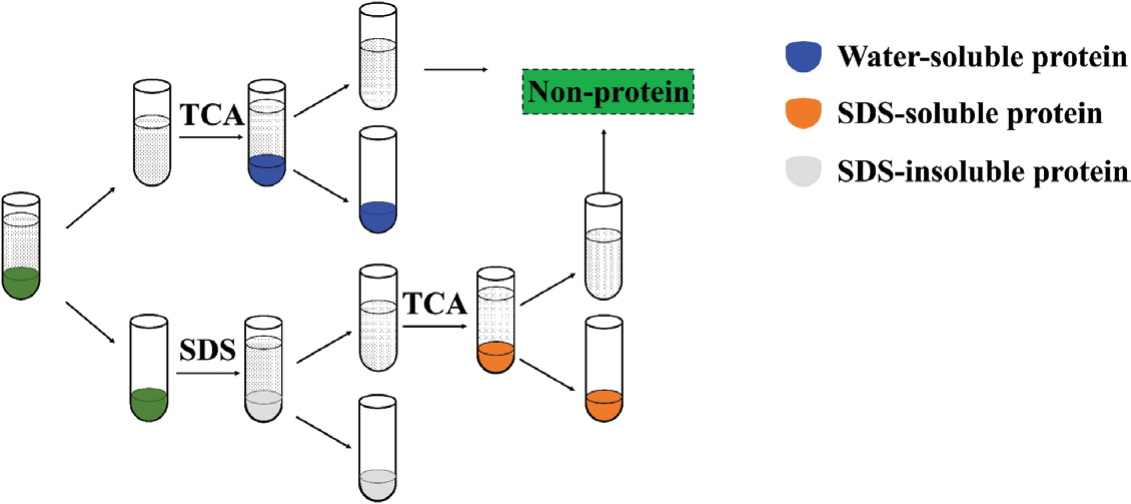
Flow chart for determination of different forms of N. TCA, trichloroacetic acid.

Finally, the third set of leaf discs was used to measure the total N content of leaves by the method of Thomas *et al.* (1967). The leaf discs were dried to constant weight and digested with H_2_SO_4_–H_2_O_2_. The N concentration in the digestion solution was analysed by continuous flow analysis. Non-protein N content (mainly inorganic N and N-containing small molecules such as amino acids, expressed as *N*_np_) was the remaining N after the removal of the above three forms of protein N (Fig. 2).

### N partitioning by function

**Fig. 3.**
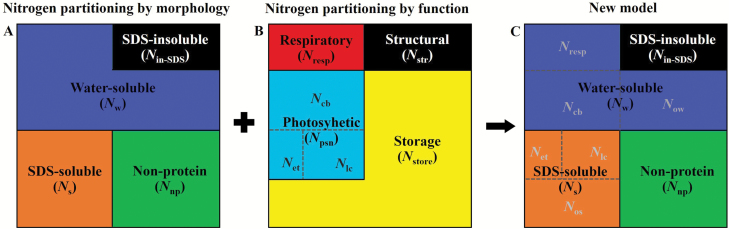
New model for distinguishing storage N forms. Leaf N partitioning by morphology (A), which is divided into SDS-insoluble protein N (N_in-SDS_), water-soluble protein N (*N*_w_), SDS-soluble protein N (*N*_s_), and non-protein N (*N*_np_). Leaf N partitioning by function (B), which is divided into structural N (*N*_str_), respiratory N (*N*_resp_), photosynthetic N [*N*_psn_; contains carboxylation (*N*_cb_), electron transfer (*N*_et_), and light capture (*N*_lc_) systems N], and storage N (*N*_store_). The remaining *N*_w_ forms with *N*_cb_ and *N*_resp_ removed is expressed as *N*_ow_; the remaining *N*_s_ with *N*_lc_ and *N*_et_ removed is expressed as *N*_os._ In this way, storage N is divided into three forms: water-soluble protein (*N*_ow_), SDS-soluble protein (*N*_os_), and non-protein (*N*_np_).

According to the LUNA model developed by Ali and Xu (Xu *et al.*, 2012; Ali *et al.*, 2016), leaf N is divided into photosynthetic N, respiration N, storage N, and structural N (Fig. 3). As described by Niinemets and Tenhunen (1997), N involved in photosynthesis (*N*_psn_) was further divided into three major parts: carboxylation system (*N*_cb_, proteins for carboxylation in the Calvin cycle); electron transport components (*N*_et_, proteins involved in electron transport); and light capture system (*N*_lc_, proteins for light capture in PSI, PSII, and other light-harvesting pigment protein complexes). Respiratory N (*N*_resp_) represents the respiratory enzymes located in the mitochondrial matrix. Storage N (*N*_store_) is the N stored in plant tissues that is not involved in any metabolic processes or structural components. Structural N (*N*_str_) is mainly used to build cell walls and is set to a fixed value (0.001 g or 0.002 g N g biomass, based on the C:N ratio from dead wood) in the model. It can be measured directly and is expressed by the SDS-insoluble protein N (*N*_in-SDS_) in this study. The calculation formulas for each N are given below.

The model assumes that the carboxylation rate is proportional to Rubisco activity. With knowledge of the specific activity of Rubisco [*V*_cr_, i.e. the maximum rate of RUBP carboxylation per unit Rubisco protein (μmol CO_2_ g^−1^ Rubisco s^−1^)], the carboxylation N content (*N*_cb_) is given by:

$$N_{cb}\;=\;\frac{V_{{}_{c,max}}}{6.25\times V_{cr}\;\times f_{Vc,max}\;}$$ (4)

where 6.25 is the coefficient of N conversion into Rubisco (g Rubisco g^−1^ N) (Jordan and Ogren, 1984); *V*_cr_ is 20.78 µmol CO_2_ g^−1^ Rubisco s^−1^ at 25 °C (Niinemets and Tenhunen 1997); and *f*_*V*c,max_ is a correction coefficient standardized to 25 °C for *V*_c,max_ by the temperature dependence function and is 0.359 at 15 °C (Supplementary Text S1).

The model assumes that N investments in electron transport is proportional to the activity of electron transport (*J*_max_), and *J*_mc_ is the maximum electron transport rate per unit of cytochrome *f* s^–1^. The electron transport N content (*N*_et_) is given by:

$$N_{et}\;=\frac{J_{max}}{8.06\times J_{mc}\times f_{Jmax}}$$ (5)

where 8.06 is the N binding coefficient for cytochrome *f* (Nolan and Smillie, 1977); *J*_mc_ is 155.65 µmol e^–^ µmol cytochrome *f* s^–1^ at 25 °C (Niinemets and Tenhunen, 1997); and *f*_*J*max_ is a correction coefficient standardized to 25 °C for *J*_max_ by the temperature dependence function and is 0.714 at 15 °C (Supplementary Text S1).

The light capture N content (*N*_lc_) is:

$$N_{lc}\;=\frac{C_{c}}{C_{B}}$$ (6)

where *C*_c_ is the leaf chlorophyll concentration (mmol m^–2^), and *C*_B_ is the ratio of chlorophyll to organic leaf N in light-harvesting components (2.15 mmol g^−1^) (Niinemets and Tenhunen, 1997).

The photosynthetic N content (*N*_psn_) is:

$$N_{\text{psn}}=N_{\text{cb}}+N_{\text{et}}+N_{\text{lc}}$$ (7)

The respiratory N content (*N*_resp_) is:

$$N_{resp}\;=\frac{R_{t}}{33.69\times f_{r}}$$ (8)

$$R_{\text{t}}=0.0\text{15}V_{\text{c},\text{max}}$$ (9)

where *R*_t_ is the leaf total respiration rate (μmol CO_2_ m^–2^ s^–1^), calculated in proportion to *V*_c,max_ (Collatz *et al.*, 1991); and 33.69 is specific N use efficiency for respiration at 25 °C (μmol CO_2_ g^–1^ N s^–1^) (Makino and Osmond, 1991); and *f*_r_ is a correction coefficient standardized to 25 °C for respiration by the temperature dependence function and is 0.522 at 15 °C (Supplementary Text S1).

The storage N content (*N*_store_) is the remaining fraction of total N content (*N*_a_) with the other components of N removed. *N*_store_ is:

$$N_{\text{store}}=N_{\text{a}}–N_{\text{psn}}–N_{\text{resp}}–N_{\text{str}}$$ (10)

where *N*_str_ is the structural N content, which is the SDS-insoluble N content (Takashima *et al.*, 2004).

### Storage N form

Combining the morphological N and functional N, storage N is divided into three forms (Fig. 3). The carboxylation system (Rubisco) and respiration proteins are mainly contained within the chloroplast matrix and mitochondrial matrix, classified as water-soluble protein (N_w_). Therefore, the remaining *N*_w_ with *N*_cb_ and *N*_resp_ removed is regarded as the water-soluble protein form of storage N (*N*_ow_) and is given by:

$$N_{\text{ow}}=N_{\text{w}}–N_{\text{cb}}–N_{\text{resp}}$$ (11)

The light capture system proteins and electron transport components are located in the thylakoid membrane of the chloroplast, classified as SDS-soluble protein (*N*_s_). The remaining *N*_s_ without *N*_lc_ and *N*_et_ is regarded as the SDS-soluble protein form of storage N (*N*_os_) and is given by:

$$N_{\text{os}}=N_{\text{s}}–N_{\text{lc}}–N_{\text{et}}$$ (12)

The non-protein N is the remaining fraction of total N, without the three previously specified fractions of N, and is regarded as the non-protein form of storage N (*N*_np_).

### Data analysis

Data were analysed statistically using SAS (SAS Institute, Cary, NC, USA). The least significant difference (LSD) test was used to assess significant differences between N treatments (*P*<0.05). Linear–plateau regressions were performed using PROC NLIN in SAS. All figures were constructed by Origin 8.0 software (OriginLab Corporation, Northampton, MA, USA).

## Results

### Leaf morphological and physiological traits

**Fig. 4.**
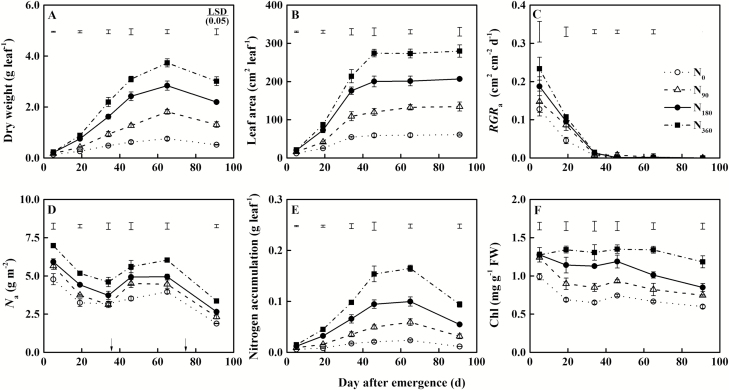
Dynamics of (A) leaf dry weight, (B) leaf area, (C) relative growth rate based on leaf area (*RGR*_a_), (D) total N content per unit leaf area (*N*_a_), (E) N accumulation, and (F) chlorophyll concentration (Chl) along with the growth days of marked leaves of *Brassica napus* L. under different N supply. The bars at the top of the graph indicate the least significant difference (LSD) among treatments (*P*<0.05). Black arrows indicate the timing of top dressing of urea. Each point represents the mean of three repetitions per treatment and bars represent the SD.

The marked sixth leaves were completely expanded ~46 d after leaf emergence, and the dry weight reached the maximum ~65 d after leaf emergence, regardless of N treatments (Fig. 4A, B). Compared with the N_0_ treatment, the maximum leaf area and maximum dry weight were increased by 103.0–364.0% and 141.3–397.3%, respectively, in response to the N supply treatments. The relative growth rate based on leaf area (*RGR*_a_) gradually decreased until the leaves fully expanded (Fig. 4C). Area-based leaf N content (*N*_a_) decreased gradually with leaf growth but slightly increased with top dressing at 35 d after leaf emergence (Fig. 4D). N accumulation gradually increased until the leaves fully expanded, and it began to decline sharply at 65 d after leaf emergence (Fig. 4E). Chlorophyll content showed a slow decline with leaf growth (Fig. 4F). *N*_a_, N accumulation, and chlorophyll content were increased with increasing rates of N fertilizer application.

### Photosynthetic parameters

**Fig. 5.**
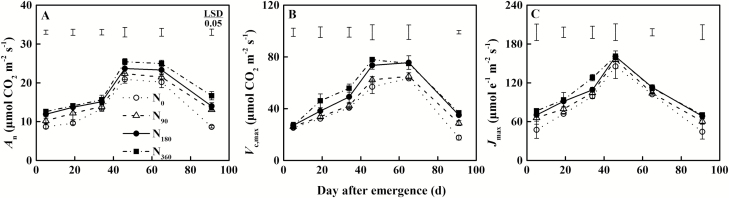
Dynamics of leaf (A) net photosynthetic rate (*A*_n_), (B) maximum carboxylation rate (*V*_c,max_), and (C) maximum electron transport rate (*J*_max_) along with the growth days of marked leaves of winter oilseed rape (*Brassica napus* L.) under different N supply. The bars at the top of the graph indicate the least significant difference (LSD) among treatments (*P*<0.05). Each point represents the mean of three repetitions per treatment and bars represent the SD.

Before the leaves fully expanded (45 d after leaf emergence), the net photosynthetic rate (*A*_n_) and the maximum carboxylation rate (*V*_c,max_) of the leaves showed no significant differences between N treatments. However, significant differences were observed after the leaves fully expanded (Fig. 5A, B). At 46 d, N supply enhanced *A*_n_ and *V*_c,max_ by 7.2–21.5% and 9.6–37.1%, respectively, relative to N_0_ treatment. The maximum electron transport rate (*J*_max_) for all N treatments reached its highest value when the leaves fully expanded, and then it decreased sharply (Fig. 5C). However, there were no significant differences in *J*_max_ among N supply treatments.

### N partitioning

**Fig. 6.**
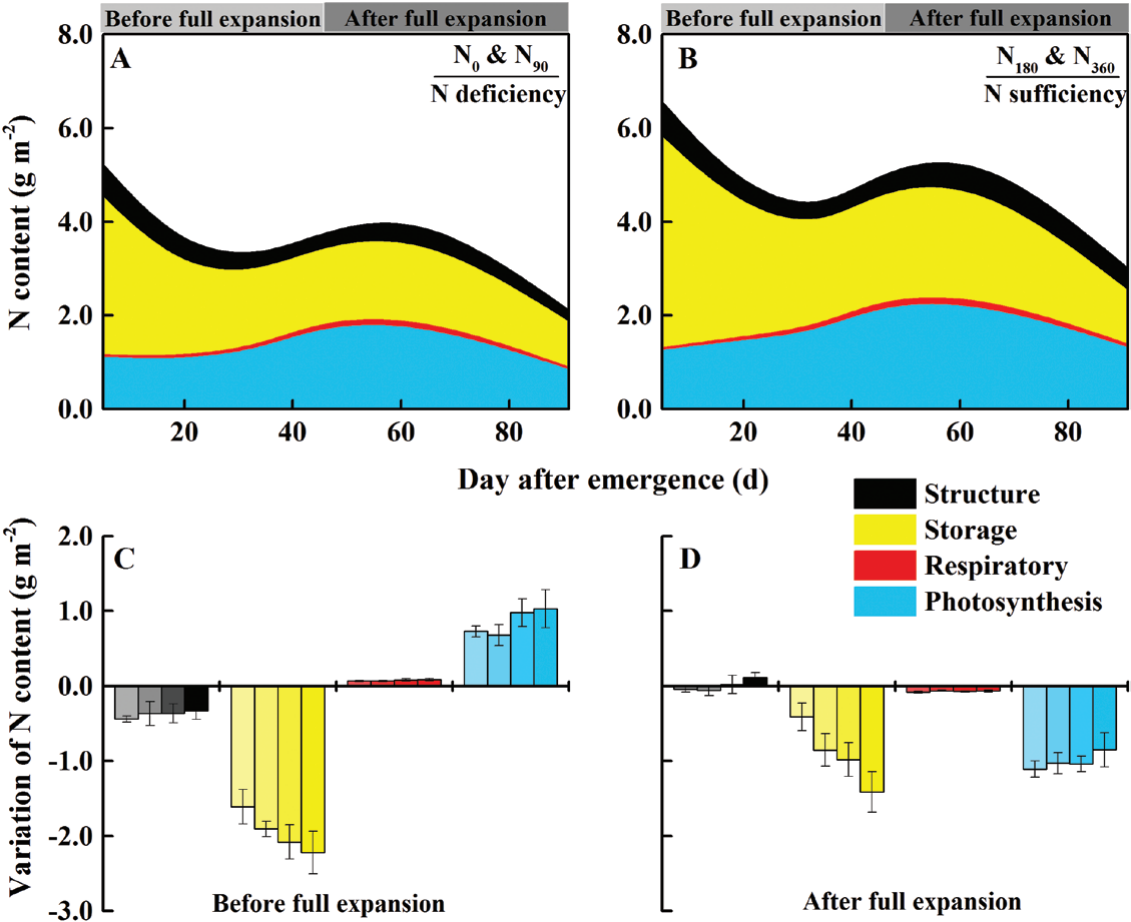
N partitioning by function of winter oilseed rape (*Brassica napus* L.) leaves along with the growth days of marked leaves under N deficiency (A) and N sufficiency (B). N_0_ and N_90_ treatments are classified as N deficiency; N_180_ and N_360_ treatments are classified as N sufficiency. The data in (A) and (B) are calculated by mean values of two N treatments, respectively. Variation of N partitioning by function of leaves (C) from emergence (S1) through full expansion (S4) and (D) from full expansion (S4) through senescence (S6) under different N supply. In the same group of histograms, the columns from left to right indicate the rates of N fertilizer application from low to high. Data are the mean of three repetitions per treatment and bars represent the SD. Leaf growth can be divided into two stages: before full expansion (S1–S3) and after full expansion (S4–S6).

N partitioning results during leaf growth under different N supply treatments based on the LUNA model (Supplementary Fig. S2) are summarized in Fig. 6A and B. The storage N content (*N*_store_) gradually decreased along with leaf expansion, and decreased by 52.4% and 47.9% under N deficiency and N sufficiency from emergence through full expansion, respectively (Fig. 6C). Meanwhile, the photosynthetic N content (*N*_psn_) gradually increased and increased by 63.7% and 80.3% under N deficiency and N sufficiency, respectively. After the leaves fully expanded, each type of N content remained stable and then decreased sharply. *N*_psn_ and *N*_store_ reduced by averages of 47.0% and 46.4%, respectively, for all N treatments from full expansion through senescence (Fig. 6D). Nevertheless, N supply retarded the decrease in *N*_psn_, which decreased by 53.4% under N deficiency and by only 41.9% under N sufficiency from full expansion through senescence. Conversely, *N*_store_ was decreased by 39.5% under N deficiency and by 51.2% under N sufficiency.

### Relationship between functional N and leaf expansion and photosynthesis

**Fig. 7.**
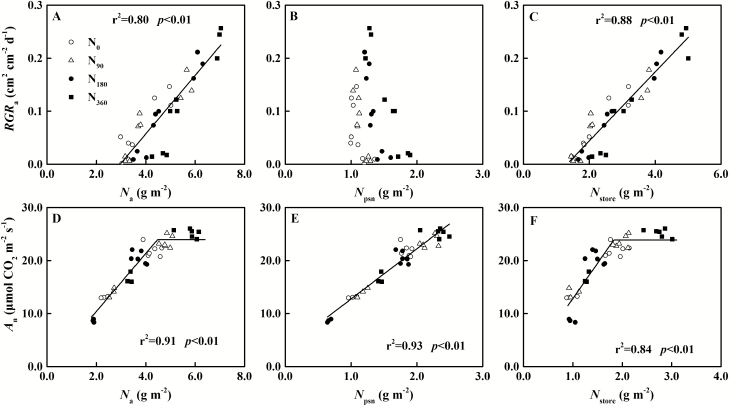
Correlations of relative growth rate (*RGR*_a_) with (A) total N content (*N*_a_), (B) photosynthetic N content (*N*_psn_), and (C) storage N content (*N*_store_) of winter oilseed rape (*Brassica napus* L.) leaves before leaf full expansion (S1–S3); correlations of net photosynthetic rate (*A*_n_) with (D) *N*_a_ and (E) *N*_psn_, and (F) *N*_store_ of leaves after leaf full expansion (S4–S6). The data from all N treatments are used together for correlation analysis and fitted by linear (A, C, E) and linear–plateau (D, F) regression, respectively.

Before the leaves fully expand, N is used primarily for leaf expansion. *RGR*_a_ was significantly positively correlated with *N*_a_ and *N*_store_, but had no significant relationship with *N*_psn_ (Fig. 7A–C). When *N*_a_ was <2.96 g m^–2^, the leaves stopped expanding. Meanwhile, the storage N content (*N*_store_) decreased to 1.32 g m^–2^. After the leaves fully expanded, *A*_n_ was positively correlated with *N*_a_, which was saturated with respect to *N*_a_. When *N*_a_ was >4.48 g m^–2^, *A*_n_ was stabilized at its highest value, 23.9 μmol m^–2^ s^–1^ (Fig. 7D). Further study revealed that the *A*_n_–*N*_psn_ relationship was a single linear regression (Fig. 7E). Meanwhile, *N*_store_ was also higher than 1.83 g m^–2^, and *N*_psn_ was maintained at 2.25 g m^–2^. When *N*_store_ was below the threshold, *A*_n_ began to decline (Fig. 7F).

### Different forms of storage N

**Fig. 8.**
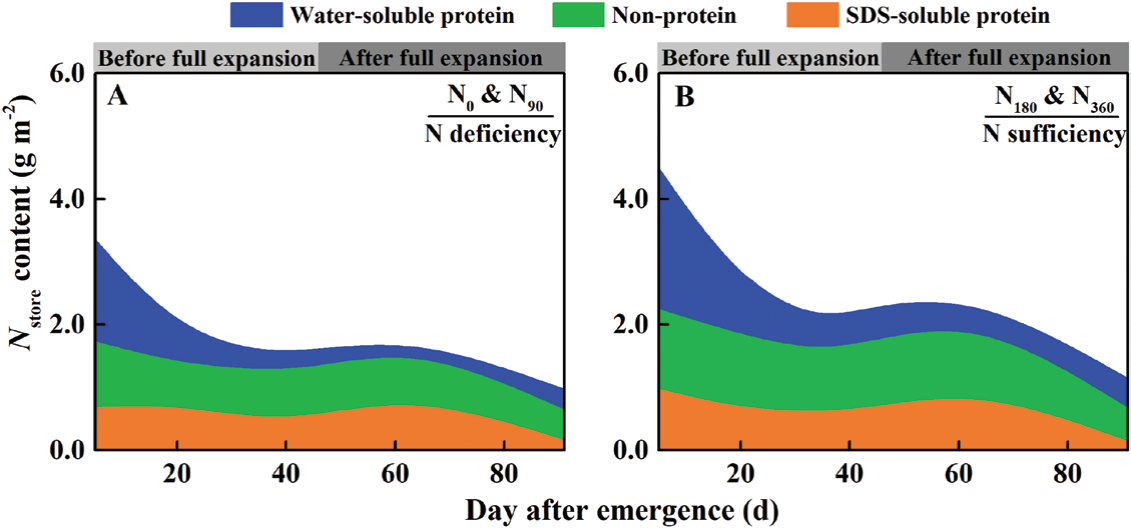
Dynamics of water-soluble protein, non-protein, and SDS-soluble protein forms of storage N during leaf growth under N deficiency (A) and N sufficiency (B). N_0_ and N_90_ treatments are classified as N deficiency; N_180_ and N_360_ treatments are classified as N sufficiency. The data in (A) and (B) are calculated by mean values of two N treatments, respectively.

The dynamics of different forms of storage N throughout the leaf growth process are presented in Fig. 8. N supply significantly increased the contents of various forms of storage N during multiple stages of leaf growth. The water-soluble protein form of storage N content (*N*_ow_) gradually decreased along with leaf expansion, and decreased by 82.4% and 75.2% under N deficiency and N sufficiency, respectively, from emergence through full expansion. Meanwhile, the non-protein form of storage N content (*N*_np_) and the SDS-soluble protein form of storage N content (*N*_os_) decreased relatively little, by averages of 19.7% and 25.0%, respectively, for all N treatments. After the leaves fully expanded, each form of storage N content remained stable, and then decreased sharply, especially *N*_np_ and *N*_os_, which decreased by 45.0% and 77.5%, respectively, on average for all N treatments. N supply intensified the decline of *N*_np_ and *N*_os_, which decreased by 51.4% and 80.9% under N sufficiency and by 36.0% and 73.2% under N deficiency.

## Discussion

### Leaf N partitioning

In C_3_ plants, more than half of leaf N is invested in the photosynthetic apparatus (Evans, 1989; Evans and Seemann, 1989). However, there are significant differences between species and environments, ranging from 30% to 70% (Evans and Poorter, 2001; Ghannoum *et al.*, 2005; Feng *et al.*, 2009; Onoda *et al.*, 2017). In addition, the structural N (tightly cross-linked in cell walls) was also found to be a considerable portion of the N in the leaves, ranging from 2.8% to 25% (Takashima *et al.*, 2004; Feng *et al.*, 2009; Harrison *et al.*, 2009; Hikosaka and Shigeno, 2009). In this study, 45.3% and 8.0% of N on average for all N treatments in fully expanded leaves were allocated to photosynthetic activities and structure, respectively, which is consistent with previous studies. The cytoplasm contains amino acids and numerous proteins that are not directly related to photosynthesis and respiration, but have not been fully quantified (Onoda *et al.*, 2017). Moreover, part of the amino acids and inorganic N are stored in the vacuole (Evans and Poorter, 2001; Funk *et al.*, 2013). In the ecological model, this fraction of N was defined as storage N, which does not participate in any metabolic processes and structural constituents. In herbaceous plants, storage N was found to be >50% of leaf N (Xu *et al.*, 2012), whereas this value was observed to be an average of 43.8% for all N treatments in this study. Although storage N calculated by the model includes the N in nucleic acids and defence substances, it cannot be ignored. Previous studies have shown that 10–15% of leaf N was allocated to nucleic acids and ribosomes (Chapin and Kedrowski, 1983; Evans and Seemann, 1989; Funk *et al.*, 2013); in some species, alkaloids and other defence substances accounted for a small proportion (Miller and Woodrow, 2008). Therefore, 20–30% of N is still not involved in any metabolism and is used as storage N. In addition, the distribution of resources between enzymes of carbon metabolism is not optimal, such as Rubisco (Makino *et al.*, 2003; Zhu *et al.*, 2007). Rubisco accounted for 20–40% of soluble protein in C_3_ plants (Evans and Seemann, 1989; Poorter and Evans, 1998; Makino *et al.*, 2003; Mu *et al.*, 2016); however, an inactivated subset of Rubisco could be considered storage N (Warren *et al.*, 2003). Recently, the chlorophyll-binding proteins Msf1 and Scp have also been regarded as storage proteins as a resource for repairing photodamaged PSII (Komenda and Sobotka, 2016; Zhao *et al.*, 2017). Therefore, distinguishing among forms of storage N is of great significance for understanding leaf growth.

### Storage N for leaf expansion and photosynthetic capacity

**Fig. 9.**
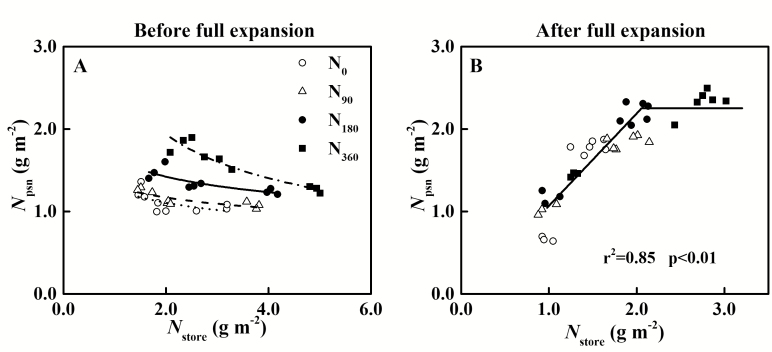
Relationship between storage N and photosynthetic N (A) before leaf full expansion (S1–S3) and (B) after leaf full expansion (S4–S6). The data in (A) are power equation fits of N_0_ (dot line), N_90_ (dash line), N_180_ (solid line), or N_360_ (dash dot line) treatment. The data in (B) are fitted by linear–plateau regression.

Storage N is mainly used for the growth of new tissue and the synthesis of photosynthetic proteins. Storage N decreased along with leaf expansion and, when *N*_store_ was <1.32 g m^-2^, the leaves stopped expanding (Fig. 7C). The construction of new tissue requires basic N supply until storage N is insufficient (Williams *et al.*, 1987; Millard, 1988; Xu *et al.*, 2012). However, the remaining *N*_store_ in leaves was higher with increasing rates of N application rate, being 1.20 g m^–2^ for N deficiency and 1.89 g m^–2^ for N sufficiency (Supplementary Fig. S3). Leaf expansion is controlled not only by N status but also by carbon status (Lattanzi *et al.*, 2005; Pantin *et al.*, 2011). Therefore, higher N reserves in the leaves may be due to carbon limitation under N sufficiency, that plays a key role in maintaining leaf photosynthetic capacity. In addition, as with the ‘potato strategy’ (Vos and Van der Putten, 1998; Vos *et al.*, 2005), photosynthetic capacity is increased at the expense of leaf area under N deficiency in winter oilseed rape (*B. napus* L.). Studies on ‘antisense’ *rbc*S plant also found that the specific leaf area was increased with decreased Rubisco (Fichtner *et al.*, 1993; Makino *et al.*, 2000). This finding demonstrated that there was a trade-off between photosynthetic N and storage N (Fig. 9). Before the leaves fully expanded, there was a significant gradient effect between *N*_store_ and *N*_psn_ under different N supply treatments (Fig. 9A). In addition, *N*_psn_ increased rapidly at the late stages of expansion compared with the previous period. The response of *N*_psn_ to *N*_store_ was saturated after the leaf fully expanded (Fig. 9B). When *N*_store_ was >2.06 g m^–2^, *N*_psn_ was maintained at a plateau value of 2.25 g m^–2^; at lower levels of *N*_store_, *N*_psn_ was followed by a linear decline.

The photosynthetic capacity was closely related to leaf N content (*N*_a_), which was also affected by various environmental factors, especially at high N levels (Rotundo and Cipriotti, 2017). In this study, when *N*_a_ reached 4.48 g m^–2^, *A*_n_ attained the highest value of 23.9 μmol CO_2_ m^–2^ s^–1^ and then no longer increased, which is consistent with previous research (Vos *et al.*, 2005; Palmroth *et al.*, 2013; Rotundo and Cipriotti, 2017). There are many reasons for this phenomenon, including CO_2_ diffusion and the kinetics of photosynthetic enzymes (especially for Rubisco) (Bernacchi *et al.*, 2002; Yamori *et al.*, 2016). Larger chloroplasts reduced the ratio of mesophyll conductance to Rubisco content under high N supply, which resulted in insufficient supply of CO_2_ in chloroplasts (Li *et al.*, 2012, 2013). Less of the Rubisco protein is enzymically active as a result of limitation of CO_2_ supply, with the rest of Rubisco acting as a storage protein (Ekman *et al.*, 1989; Warren, 2004; Eichelmann *et al.*, 2005; Gupta *et al.*, 2015). Numerous studies have been devoted to reducing the amount of Rubisco and improving its efficiency to achieve higher resource use efficiency (Suzuki *et al.*, 2007, 2009; Carmo-Silva *et al.*, 2015). However, there was limited N inflow after the leaf fully expanded (Makino *et al.*, 1984; Imai *et al.*, 2005). At that stage, maintaining high photosynthetic performance of the leaf is the key to improving dry matter accumulation. The results of this study revealed that when *N*_store_ exceeded 1.83 g m^–2^, the leaf could maintain *N*_psn_ and *A*_n_, whereas, below the threshold, *A*_n_ declined (Figs 7F, 9B). As Rubisco is in a dynamic balance of synthesis and degradation (Imai *et al.*, 2005; Irving and Robinson, 2006), maintaining photosynthetic proteins requires a source of N, which is supplied by storage N (Raven, 2011).

The turnover and amount of storage N are also related to the source and sink strength at the whole-plant level (Lehmeier *et al.*, 2013). Storage N in leaves will be increased by N supply (Gloser, 2005; Wyka *et al.*, 2016), which also increases the risk of high N residues (Yasumura and Ishida, 2011; Avice and Etienne, 2014). N remobilization was inhibited under excessive N supply, resulting in N loss including ammonia volatilization during leaf senescence (Schjoerring *et al.*, 1998). Under N deficiency, there is high demand for N in the new leaves; this reduces the size of the storage N pool and leads to early senescence in the mature leaves. Therefore, a certain amount of storage N can delay leaf senescence and reduce N loss.

### Different forms of storage N

**Fig. 10.**
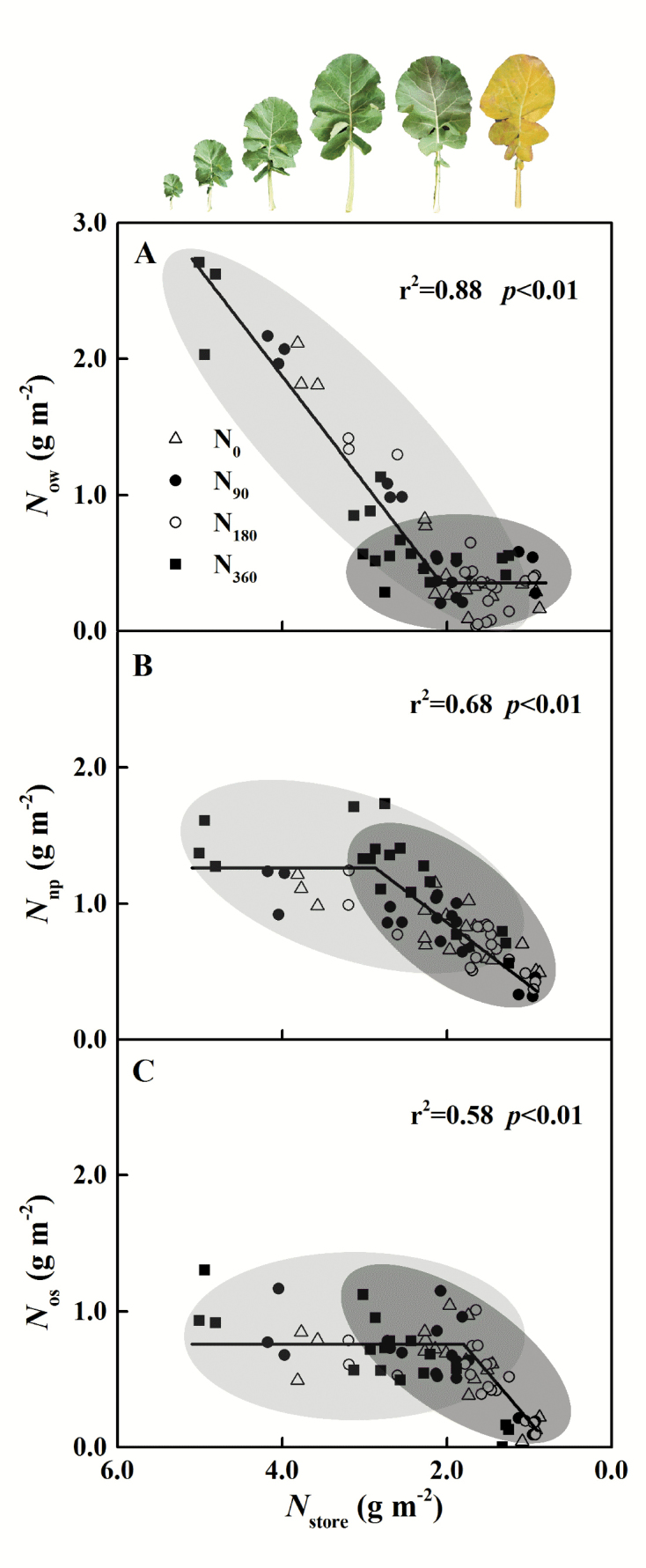
Correlations of storage N content (*N*_store_) with (A) the water-soluble protein form of storage N content (*N*_ow_), (B) the non-protein form of storage N content (*N*_np_), and (C) the SDS-soluble protein form of storage N content (*N*_os_) during leaf growth. The data before leaf full expansion (S1–S3) are covered by the light grey oval; the data after leaf full expansion (S4–S6) are covered by the dark grey oval. The data in (A)–(C) are fitted by linear–plateau regression.

Different forms of storage N supplying leaf growth showed the sequence in terms of degradation. Water-soluble proteins were more susceptible to degradation than other types of proteins, such as ‘membrane proteins’ (SDS-soluble proteins, mostly in the thylakoid membrane) (Eichelmann *et al.*, 2005; Yasumura *et al.*, 2007). This study demonstrated that when *N*_store_ was >2.28 g m^–2^ (Fig. 10A), which occurred during the period of rapid leaf expansion, the water-soluble protein form of storage N was the N source for leaf expansion and photosynthetic protein synthesis. As the leaves continued to grow until fully expanded, the non-protein form of storage N began to supply leaf expansion (Fig. 10B). After the leaves fully expanded, storage N was utilized to maintain leaf photosynthesis. At that stage, water-soluble protein and non-protein forms of storage N were the main supplies of N. This was followed by the late growth stage. Once *N*_store_ decreased to 1.89 g m^–2^, the SDS-soluble protein form of storage N began to be degraded to supply leaf photosynthesis (Fig. 10C).

### Model limitations

The source of error in estimating storage N content based on the model was mainly the estimation of photosynthetic N, which was calculated using the methods introduced by Niinemets and Tenhunen (1997). This method not only considers the carboxylation ability and electron transport ability but also uses chlorophyll content as a variable in the formula. The reliability of the model has been verified by other studies (Frak *et al.*, 2002; Grassi *et al.*, 2002; Trouwborst *et al.*, 2011; Chen *et al.*, 2014). These studies involve the effects of species, light, CO_2_ concentration, and N nutrition on photosynthetic N partitioning. These studies have also indicated that the model underestimated Rubisco content relative to the content of Rubisco *in vitro* quantification (Warren and Adams, 2001, Warren *et al.*, 2003; Bahar *et al.*, 2017), and the difference between the two was due to inactivation of Rubisco as N store. Apparently, it has been classified as storage N in this model. However, some limitations regarding this model remain. For example, it does not account for the limitation of triose phosphate utilization on carboxylation and electron transfer (Harley *et al.*, 1992; Niinemets and Tenhunen, 1997), especially for winter crops, which led to the underestimation of photosynthetic N under low temperature. In addition, the determination of the parameters (*H*_a_, *H*_d_, *S*_v_, and *c*) in the temperature dependence function of *V*_c,max_ (*V*_cr_) and *J*_max_ (*J*_mc_) affects the accuracy of the model, and there are differences among species, especially for *H*_a_ (Kattge and Knorr, 2007). Sensitivity analysis showed that *V*_cr_ and *J*_mc_ were most sensitive to variation in *H*_a_ and *c* (Supplementary Text S1).

### Conclusions

Storage N content (*N*_store_) increased with the increase in N supply and decreased gradually along with leaf expansion. The relative growth rate based on leaf area (*RGR*_a_) was determined by storage N during leaf expansion, not photosynthetic N. When *N*_store_ decreased to a threshold, the leaves stopped expanding. The water-soluble protein form of storage N was the main N source for leaf expansion. After the leaves fully expanded, the net photosynthetic rate (*A*_n_) followed a linear–plateau response to *N*_store_, with *A*_n_ stabilizing at the highest value above a threshold and declining below the threshold. However, the decline in the SDS-soluble protein form of storage N was the main factor leading to the decline in *A*_n_. Hence, for leaf N economy, storage N is used for co-ordinating leaf expansion and photosynthetic capacity. Turnover between storage N and photosynthetic N could maintain a high photosynthetic rate. N supply can increase *N*_store_, thereby promoting leaf growth and biomass.

## Supplementary data

Supplementary data are available at *JXB* online.

**Fig. S1.** Photograph of marked leaves in winter oilseed rape (*Brassica napus* L.) along with the growth days under N supply.

**Fig. S2.** N partitioning by function of winter oilseed rape (*Brassica napus* L.) leaves along with the growth days of marked leaves under N supply.

**Fig. S3.** Relationship between the relative growth rate and storage N under N supply.

**Text S1.** Temperature-dependent functions of model parameters.

Supplementary Figures

Supplementary File

## Author contributions

JL, TR, and TL designed the research. TL conducted the field experiments and collected the data. TL and TR performed the analysis and wrote the manuscript. JL, TR, PW, and RC revised the manuscript.

## Abbreviations:

*A*_n_: net photosynthetic rate
*C*_B_: ratio of chlorophyll to nitrogen in light-harvesting components
*C*_c_: leaf chlorophyll concentration
*C*_i_: intercellular CO_2_ concentration
*f*_*_J_*max_: correction coefficient standardized to 25 °C for *J*_max_
*f*_r_: correction coefficient standardized to 25 °C for respiration
*f*_*_V_*c,max_: correction coefficient standardized to 25 °C for *V*_c,max_
*J*_max_: maximum electron transport rate
*J*_mc_: maximum electron transport rate per unit cytochrome *f* s^–1^
*K*_c_: Rubisco Michaelis constant for CO_2_
*K*_o_: Rubisco Michaelis constant for O_2_
LUNA: leaf utilization of nitrogen assimilation model
*N*_cb_: nitrogen content of carboxylation system
*N*_et_: nitrogen content of electron transport components
*N*_in-SDS_: nitrogen content of SDS-insoluble protein
*N*_lc_: nitrogen content of light capture system
*N*_np_: nitrogen content of non-protein
*N*_os_: the remaining *N*_s_ with *N*_lc_ and *N*_et_ removed
*N*_ow_: the remaining *N*_w_ with *N*_cb_ and *N*_resp_ removed
*N*_psn_: photosynthetic nitrogen content
*N*_resp_: respiratory nitrogen content
*N*_s_: nitrogen content of SDS-soluble protein
*N*_store_: storage nitrogen content
*N*_str_: structural nitrogen content
*N*_w_: nitrogen content of water-soluble protein
O: concentration of oxygen in air
*R*_d_: mitochondrial respiration rate in the light
*RGR*_a_: relative growth rate based on leaf area
*R*_t_: leaf total respiration rate
SDS: sodium dodecyl sulfate
*V*_c,max_: maximum carboxylation rate
*V*_cr_: specific activity of Rubisco
VSP: vegetative storage protein
Γ*: CO_2_ compensation point.
