# Supplementary material for: Storage nitrogen co-ordinates leaf expansion and photosynthetic capacity in winter oilseed rape
Source: J Exp Bot. 2018 Apr 12;69(12):2995–3007. doi: 10.1093/jxb/ery134 (PMC5972566; doi:10.1093/jxb/ery134)
Supplement: Supplementary Figures [file ery134_suppl_supplementary_figures.pdf]

Supplementary Figures

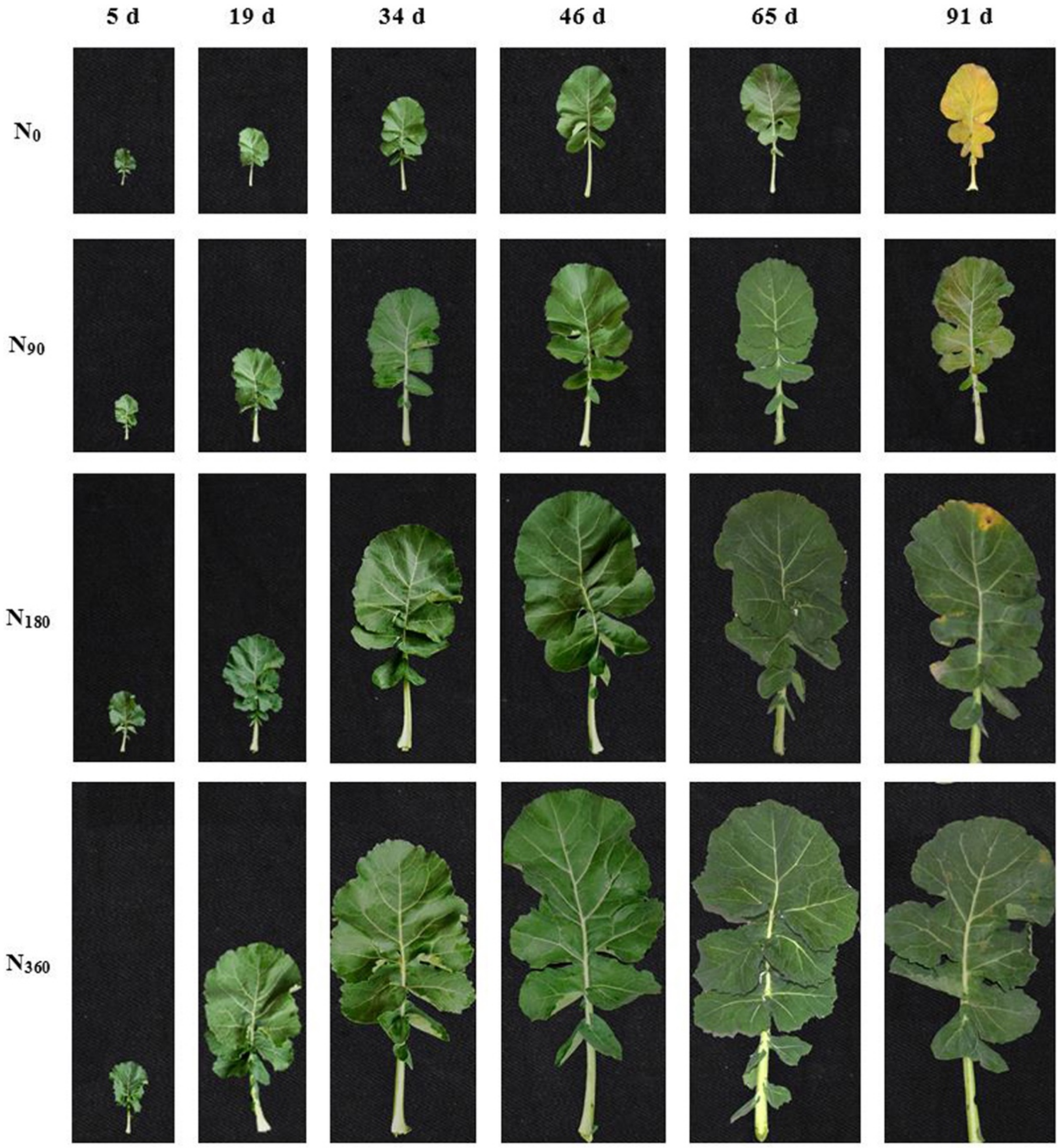

Fig. S1 Photograph of marked leaves in winter oilseed rape (Brassica napus L.) along with the growth days under N supply.

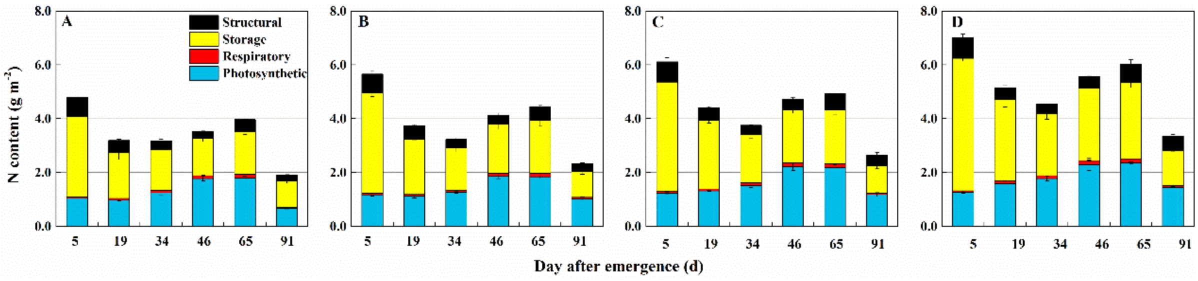

Fig. S2 N partitioning by function of winter oilseed rape (Brassica napus L.) leaves along with the growth days of marked leaves of winter oilseed rape (Brassica napus L.) under N_0 (A), N_{90} (B), N_{180} (C) and N_{360} (D) treatments. Different types of N showed a significant gradient increase with the increase of N application amount, and the regularity of different types of N reduction for each nitrogen treatment with the growth process of leaves are similar. Therefore, N_0 and N_{90} treatments are classified as N deficiency; N_{180} and N_{360} treatments are classified as N sufficiency. It has been summarized in Fig. 6A and 6B.

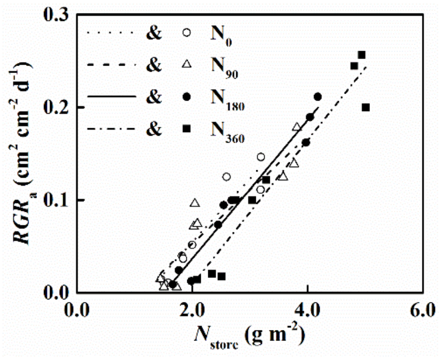

Fig. S3 Relationship between the relative growth rate and storage N under N supply.
