# Supplementary material for: Storage nitrogen co-ordinates leaf expansion and photosynthetic capacity in winter oilseed rape
Source: J Exp Bot. 2018 Apr 12;69(12):2995–3007. doi: 10.1093/jxb/ery134 (PMC5972566; doi:10.1093/jxb/ery134)
Supplement: Supplementary File [file ery134_suppl_supplementary_file_s1.pdf]

Temperature dependence functions of model parameters

K_c, K_o and R_d are Michaelis-Menten constants for carboxylation, oxygenation, and mitochondrial respiration in light, respectively. According to Bernacchi *et al.*(2001), the temperature dependence functions for K_c, K_o and R_d are given by:

$$K_o(T)=27\,840e^{[(\frac{36\,380}{RT_0})(1-\frac{T_0}{T})]} \quad \text{(S1)}$$

$$K_c(T)=40.49e^{[(\frac{79\,430}{RT_0})(1-\frac{T_0}{T})]} \quad \text{(S2)}$$

$$f_r(T)=e^{[(\frac{46\,390}{RT_0})(1-\frac{T_0}{T})]} \quad \text{(S3)}$$

where the correction coefficient are standardized to 25 °C (i.e. 298.15 K); R is the universal gas constant (8.314 J mol^{-1} K^{-1}); T is the leaf temperature (K) and the reference temperature (T_0) is 298.15 K.

Following Niinemets *et al.*(1997), the temperature dependence functions of the specific activity of Rubisco (V_{cr}) and the potential rate of photosynthetic electron transport per unit cytochrome *f* (J_{mc}) are described below. And the temperature dependence functions of $V_{c,max}$ and J_{max}, which is based on Medlyn *et al.* (2002) but modified by Kattge and Knorr (2007), are given by:

$$V_{cr}\,(J_{mc}) = \frac{e^{(c-\frac{\Delta H_a}{RT})}}{1+e^{(\frac{\Delta ST-\Delta H_d}{RT})}} \quad \text{(S4)}$$

$$V_{c,max}\,(J_{max}) = \frac{(1+e^{(\frac{\Delta ST_0-\Delta H_d}{RT_0})})e^{[(\frac{\Delta H_a}{RT_0})(1-\frac{T_0}{T})]}}{1+e^{(\frac{\Delta ST-\Delta H_d}{RT})}} \quad \text{(S5)}$$

Where ΔH_a, ΔH_d, ΔS and c are an activation energy, a deactivation energy, an entropy term and a scaling constant, respectively. The values for V_{cr} and $V_{c,max}$ are 74 000 (J

mol^{-1}), 203 000 (J mol^{-1}), 645 (J K^{-1} mol^{-1}) and 32.9, respectively; The values for J_{mc} and J_{max} are 24 100 (J mol^{-1}), 564 150 (J mol^{-1}) and 1 810 (J K^{-1} mol^{-1}) and 14.77, respectively.

Sensitivity analysis of V_{cr}, $V_{c,max}$, J_{mc} and J_{max} to changes in proportion of ΔH_a, ΔH_d, ΔS and c as following:

Table 1 Sensitivity analysis of V_{cr} and $V_{c,max}$ to changes in proportion of ΔH_a, ΔH_d, ΔS and c.

Variation	V_{cr} at 25 ℃				$V_{c,max}$ standardized to 25 ℃ at 15 ℃		
	ΔH_a	ΔH_d	ΔS	c	ΔH_a	ΔH_d	ΔS
5.0%	4.7	21.0	12.8	107.6	0.341	0.355	0.563
2.5%	9.8	21.0	19.3	47.3	0.350	0.355	0.386
0.0%	20.8	20.8	20.8	20.8	0.359	0.359	0.359
-2.5%	43.8	19.1	21.0	9.1	0.369	0.389	0.356
-5.0%	92.4	11.7	21.0	4.0	0.378	0.607	0.355

Table 2 Sensitivity analysis of J_{mc} and J_{max} to changes in proportion of ΔH_a, ΔH_d, ΔS and c.

Variation	J_{mc} at 25 ℃				J_{max} standardized to 25 ℃ at 15 ℃		
	ΔH_a	ΔH_d	ΔS	c	ΔH_a	ΔH_d	ΔS
5.0%	95.7	155.7	41.8	325.7	0.702	0.714	2.655
2.5%	122.1	155.7	153.8	225.2	0.708	0.714	0.722
0.0%	155.6	155.6	155.6	155.6	0.714	0.714	0.714
-2.5%	198.5	153.3	155.7	107.6	0.720	0.724	0.714
-5.0%	253.1	28.5	155.7	74.4	0.726	3.890	0.714

Literature

Bernacchi CJ, Singsaas EL, Pimentel C, Portis Jr AR, Long SP. 2001. Improved temperature response functions for models of Rubisco-limited photosynthesis. *Plant, Cell & Environment* 24:253-259.

Medlyn BE, Dreyer E, Ellsworth D, Forstreuter M, Harley PC, Kirschbaum MU, Le Roux X, Montpied P, Strassemeyer J, Walcroft A, Wang K. 2002. Temperature response of parameters of a biochemically based model of photosynthesis. II. A review of experimental data. *Plant, Cell & Environment* 25:1167-1179.

Niinemets Ü, Tenhunen JD. 1997. A model separating leaf structural and physiological effects on carbon gain along light gradients for the shade- tolerant species *Acer saccharum*. *Plant, Cell & Environment* 20: 845-866.

Kattge J, Knorr W. 2007. Temperature acclimation in a biochemical model of photosynthesis: a reanalysis of data from 36 species. *Plant, cell & environment* 30: 1176-1190.
